# Simulation of Wire Arc Additive Manufacturing in the Reinforcement of a Half-Cylinder Shell Geometry

**DOI:** 10.3390/ma16134568

**Published:** 2023-06-24

**Authors:** Xiao Fan Zhao, Avelino Zapata, Christian Bernauer, Siegfried Baehr, Michael F. Zaeh

**Affiliations:** Institute for Machine Tools and Industrial Management (iwb), TUM School of Engineering and Design, Technical University of Munich, Boltzmannstr. 15, 85748 Garching, Germany; avelino.zapata@iwb.tum.de (A.Z.); christian.bernauer@iwb.tum.de (C.B.); siegfried.baehr@iwb.tum.de (S.B.); michael.zaeh@iwb.tum.de (M.F.Z.)

**Keywords:** wire arc additive manufacturing, directed energy deposition, finite element analysis, simulation, deposition sequence

## Abstract

Wire arc additive manufacturing (WAAM) is an additive manufacturing process based on gas metal arc welding. It allows the fabrication of large-volume metal components by the controlled deposition and stacking of weld beads. Next to the near-net-shape manufacturing of metal components, WAAM is also applied in the local reinforcement of structural parts, such as shell geometries. However, this procedure can lead to undesired thermally induced distortions. In this work, the distortion caused by the WAAM reinforcement of half-cylinder shell geometries was investigated through experiments and transient thermo-mechanical finite element simulations. In the experiments, the weld beads were applied to the specimen, while its thermal history was measured using thermocouples. The developing distortions were registered using displacement transducers. The experimental data were used to calibrate and validate the simulation. Using the validated model, the temperature field and the distortions of the specimens could be predicted. Subsequently, the simulation was used to assess different deposition patterns and shell thicknesses with regard to the resulting part distortions. The investigations revealed a non-linear relation between shell thickness and distortion. Moreover, the orientation and the sequence of the weld beads had a significant impact on the formation of distortion. However, those effects diminished with an increasing shell thickness.

## 1. Introduction

Wire arc additive manufacturing (WAAM) is a rapidly emerging technology that has attracted considerable attention in the manufacturing industry. WAAM is a directed energy deposition (DED) technology that uses an arc welding process to manufacture metal parts layer by layer. In the process zone, a welding wire is melted by an electric arc and deposited onto a substrate or a previous layer. Compared to other metal additive manufacturing (AM) technologies, such as powder bed fusion, WAAM has demonstrated several advantages, including high deposition rates, large build volumes, a high degree of energy efficiency, and relatively low investment costs [1].

One promising field of application for WAAM is in the chemical engineering industry [2]. In this industry, various components consist of shells with mostly cylindrical geometries, such as pipes, pressure vessels, and tanks. During the design phase, the wall thickness of the entire part is chosen based on the required thickness at the most critical location [3,4]. Parts manufactured with conventional manufacturing processes typically have a constant wall thickness, which results in a significant material waste due to oversizing. In order to address this issue, WAAM can be used to reinforce parts only locally and increase the component strength at critical sections. This approach allows for the selective application of material in specific areas. As a result, it has the potential to significantly reduce the overall material usage while keeping the original functionality, making it a more sustainable and cost-effective approach.

However, one of the challenges of using WAAM to reinforce cylindrical shell geometries is the inherent formation of thermally induced part distortions [2]. Steep thermal gradients during the welding process drive the formation of residual stresses [5]. The WAAM part experiences local heating and cooling cycles of varying amplitudes, resulting in complex residual stress distributions. These residual stresses, if exceeding the local ultimate tensile strength, can result in cracking, whereas if exceeding only the local yield strength result in plastic deformation [6]. This phenomenon is commonly referred to as thermal distortion or thermal warpage and presents potential risks to the functionality of the part.

In order to investigate these effects, finite element analysis can be used to model the behavior of the part during the WAAM process and to predict potential issues in advance. This can include analyzing the thermal and mechanical behavior of different parts as well as evaluating the impact of various deposition patterns on the final product.

In the case of welding, it was demonstrated that the welding sequence of multi-weld bead parts affects the shape and the magnitude of the distortion [7,8]. Similarly, it was shown that part distortion varies significantly in WAAM depending on the deposition pattern, as observed both in experiments [9] and simulations [10,11,12]. Additionally, it was found that the part geometry has a significant impact on the resulting residual stresses and distortions [13]. The findings in the current literature reveal insights into the impact of deposition pattern and the part geometry on the distortion of planar substrate plates. However, these investigations did not include variations in the substrate geometry, such as curved substrates or substrates of different thicknesses, which are also relevant for industrial applications.

This paper reports the investigation of the impact of the deposition pattern and the shell thickness on the part distortion in the WAAM process for the reinforcement of a half-cylinder shell geometry using finite element analysis. Firstly, a thermo-mechanical process simulation was calibrated and validated using measurements. Secondly, the simulation was used to calculate the part distortion for various configurations of the deposition pattern and the shell thickness. Lastly, the simulation results were analyzed and discussed.

## 2. Specimen Geometry

**Figure 1:**
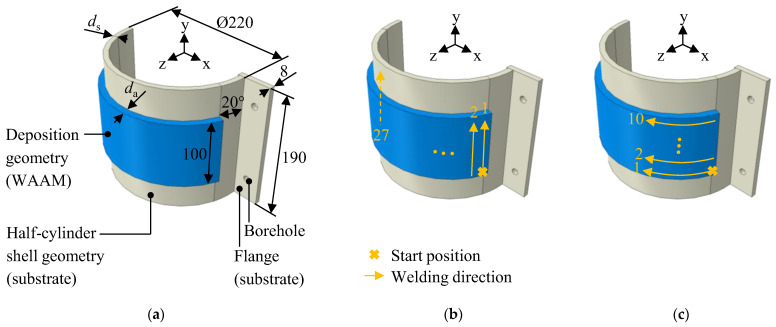
(**a**) Schematic representation of the specimen geometry; all measurements are given in mm; (**b**) schematic drawing of the axial deposition pattern; (**c**) schematic drawing of the tangential deposition pattern.

The specimen geometry comprises the substrate geometry and the deposition geometry, as shown in Figure 1a. The substrate geometry consists of a half-cylinder shell and a rectangular flange. The flange is welded to one side of the half-cylinder shell and features two boreholes used for positioning and clamping the specimen.

**Figure 2:**
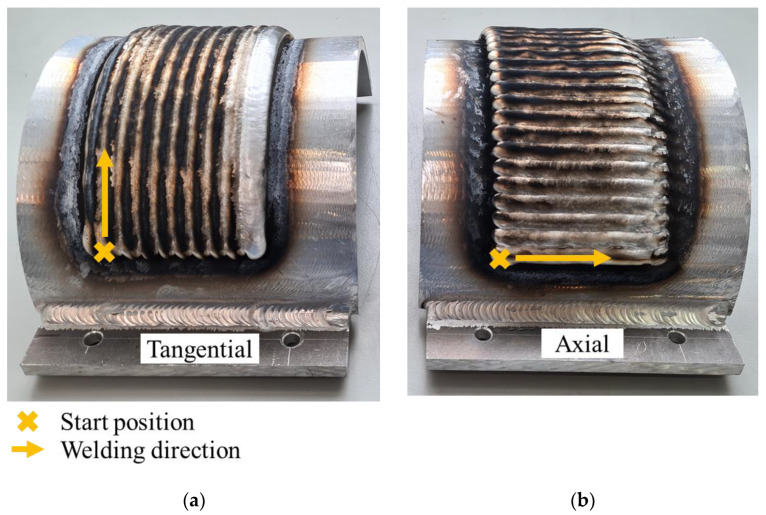
Three-layered specimens manufactured using (**a**) the tangential and (**b**) the axial deposition pattern.

Multiple versions of the geometry were analyzed in this study, considering variations in the shell thickness (*d*_s_) and the number of deposition layers. The investigated shell thicknesses were 8 mm, 10.5 mm, 18 mm, and 30 mm. The number of deposited layers was either 1 or 3, which resulted in a deposition geometry thickness (*d*_a_) of approximately 3 mm or 9 mm, respectively. Furthermore, two different deposition patterns were examined: the axial deposition pattern, in which the weld beads were oriented parallel to the half-cylinder’s axis (see Figure 1b), and the tangential deposition pattern, in which the weld beads were applied tangentially to the half-cylinder’s axis (see Figure 1c). The two deposition patterns have variations in the number and the length of the beads, as well as differences in the waiting times between consecutive beads. In the case of the axial deposition pattern, each layer comprised 27 weld beads with a length of 10 mm each. In contrast, in the case of the tangential deposition pattern, each layer comprised 10 weld beads with a length of approximately 270 mm each. Thus, the axial deposition pattern features shorter beads. However, more beads are required to complete a full layer compared to the tangential deposition pattern. For both deposition patterns, the process speed was 5 mm/s and the waiting time between two subsequent weld beads was adjusted so that each full layer took around 60 min to complete. Figure 2 illustrates three-layered specimens manufactured using the tangential (see Figure 2a) and the axial (see Figure 2b) deposition pattern.

## 3. Experimental Setup

**Figure 3:**
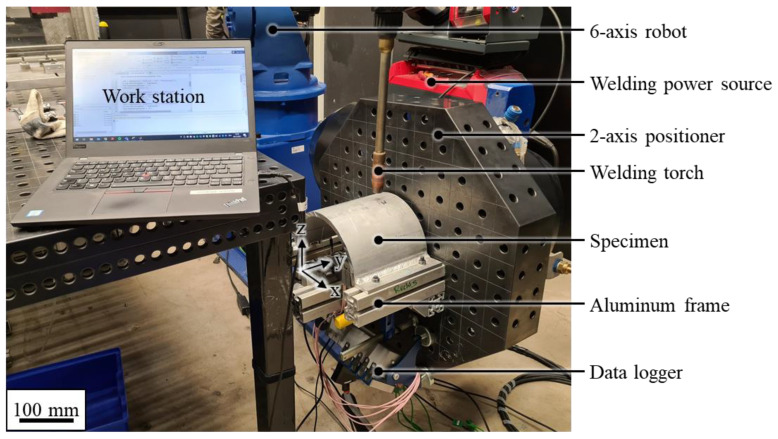
WAAM test stand inside the robotic welding chamber.

The experimental setup is shown in Figure 3. The WAAM test stand comprised several components. The kinematics of the test stand were realized using a 6-axis robotic arm (MH24 robot with a DX200 controller, Yaskawa Europe GmbH, Hattersheim am Main, Germany) and a 2-axis positioner (DK250, Yaskawa Europe GmbH, Hattersheim am Main, Germany). The welding system consisted of a welding power source (TPS 400i, Fronius International GmbH, Wels, Austria), a welding torch attached to the robotic arm, and an octagonal welding table mounted to the positioner. During a welding operation, the 2-axis positioner was used for the rotation around the *y*-axis, while the 6-axis robotic arm was used for the translation in the y- and z-directions. The specimens were clamped to the welding table of the 2-axis positioner through an aluminum frame. The position of the specimens was adjusted so that the axis of the half-cylinder shell aligned with the rotation axis of the positioner. This setup enabled the welding torch to remain perpendicular to the cylindrical shell geometry throughout all the experiments, and thus ensured comparable welding conditions.

The study employed two different Fronius welding processes: the Fronius CMT and the Fronius PMC Mix. The first layer of each specimen was welded using the more powerful Fronius PMC Mix process to establish a strong bond between the WAAM part and the substrate material. For the remaining layers, the lower-power Fronius CMT process was utilized. The welding parameters are given in Table 1.

The substrate material used in the experiments was AA 5083 (Linde GmbH, Pullach, Germany), while the wire material used was AA 5183 (Safra S.P.A, Travagliato, Italy) with a diameter of 1.2 mm. The shielding gas for the welding process was Argon 4.6 (Linde GmbH, Pullach, Germany). The chemical compositions of the substrate [14] and the wire (according to the supplier) are given in Table 2.

**Figure 4:**
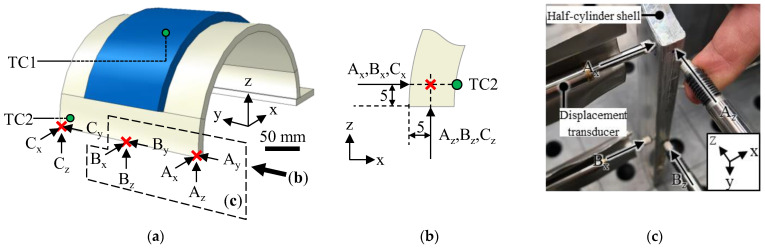
(**a**) Placement of thermocouples and displacement transducers during the experiments; the thermocouples were positioned on the inner surface of the half-cylinder shell; (**b**) detailed positioning of the displacement transducers relative to the half-cylinder shell; all measurements are given in mm; (**c**) photo of the experimental setup.

During the experiments, the thermal history and the distortion of the half-cylinder shell geometries were recorded, as indicated in Figure 4. The thermal data acquisition during the experiments was accomplished using a data logger (NI 9213 Module, National Instruments Corp, Austin, TX, USA) in connection with thermocouples (Type N, TC Mess-und Regeltechnik GmbH, Mönchengladbach, Germany) attached to the inside of the half-cylinder shell at TC1 and TC2. The sampling rate for thermal measurements was 4 Hz. In addition to the thermal measurements, the specimen disortion was measured along the vectors A_x_, A_z_, B_x_, and B_z_ using four displacement transducers (SPECTRO ST 1200, Haidenhain GmbH, Traunreut, Germany) aligned to register micrometer-scale length changes in the respective directions. The displacements along the vectors A_y_, B_y_, C_x_, C_y_, and C_z_ were only measured in the simulation results. The selection of the measuring positions was based on the anticipated maximum distortion at these locations, as they were situated at the farthest points from the specimen’s fixture. The sampling rate for distortion measurements was 1 Hz.

## 4. Simulation Setup

### 4.1. Model

A transient simulation using a weakly coupled thermo-mechanical finite element simulation was employed to model the WAAM process. The software used was Abaqus FEA version 2020 (Dassault Systèmes SE, Vélizy-Villacoublay, France). The simulation was carried out in two stages. The first stage involved the thermal simulation yielding the thermal history of the specimen during and after the WAAM process. The second stage was the mechanical simulation, in which the acquired thermal simulation results were imported as time-dependent field conditions. In this manner, the previously calculated thermal history was considered in the mechanical simulation, where it initiated residual stresses and distortions. The time step sizes used in both simulations were controlled by the solver with a maximum step size of 1 s. The effects of fluid flow in the melt pool were neglected in the simulation model [15].

**Figure 5:**
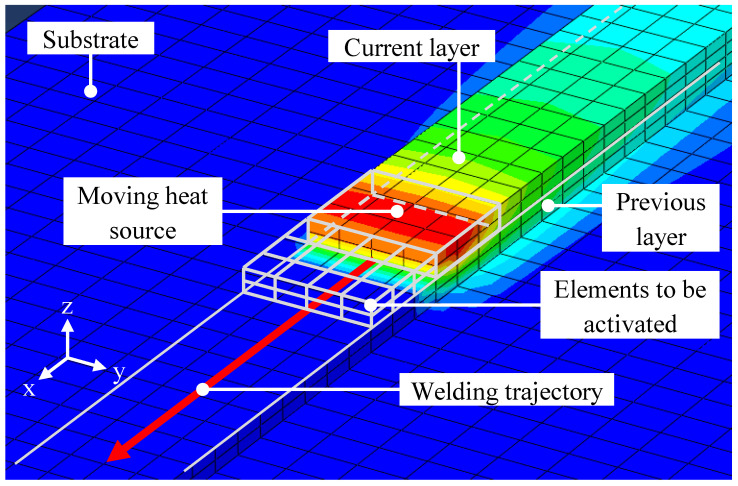
Thermo-mechanical finite element simulation environment.

The simulative approach is mainly based on the model in [16] and the simulation environment is shown in Figure 5. A cuboid heat source was used to model the heat input through the electric arc. As it traveled along a given welding trajectory, quiet elements were activated along its path [17]. A comma-separated value (CSV) file was used to communicate the welding trajectory to the simulation software. Each line within the file represented a distinct point in space and time, defining the position of the welding torch. This method facilitated the simulation of multi-layer parts.

The governing equation for heat transfer is expressed as:

(1) $$\rho \left(T\right)c_{p}\left(T\right)\frac{\partial T}{\partial t}-\nabla \left(\lambda \left(T\right)\nabla T\right)=q\left(x,t\right).$$

Here, T represents the temperature, t denotes the temporal variable, ∇ is the nabla operator, and x refers to the spatial coordinate. The variables ρ(T), $c_{p}\left(T\right)$, and λ(T) denote the temperature-dependent density, specific heat capacity, and thermal conductivity, respectively. The variable q(x,t) represents the time- and space-dependent internal cuboid heat source given by:

(2) $$q\left(x,t\right)=\eta \frac{P\left(x,t\right)}{hwl},$$

where P(x,t) denotes the power output of the welding power source at a specific time and location, η represents the heat source efficiency, and h, w, and l denote the height, width, and length of the heat source, respectively. While more refined heat source shapes have been proposed in the literature, such as the Goldak double-ellipsoid heat source [18] and the annular heat source [19], these models were designed to capture the melt pool shape and require a high temporal and spatial resolution in the simulations. For thermo-mechanical simulations focusing on distortions at a part scale, a computationally less expensive cuboid heat source is sufficient [15]. The thermal loss through convection was modeled using the convection equation:

(3) $$Q=kA\left(T_{1}-T_{2}\right).$$

Here, Q denotes the heat transfer rate, k refers to the heat transfer coefficient, A denotes the surface area where the heat transfer is taking place, $T_{1}$ represents the temperature of the solid surface, and $T_{2}$ is the temperature of the surrounding fluid. The thermal loss through radiation was neglected due to the low emission coefficient of aluminum. In the subsequent mechanical simulation, the stress equilibrium is given as:

(4) ∇σ+b=0.

Here, σ is the stress tensor and b is the body force vector. Neglecting strain resulting from phase transformations, the total strain ε is given as:

(5) $$\varepsilon =\varepsilon_{\mathrm{el}}+\varepsilon_{\mathrm{pl}}+\varepsilon_{\mathrm{th}},$$

where $\varepsilon_{\mathrm{el}}$, $\varepsilon_{\mathrm{pl}}$, and $\varepsilon_{\mathrm{th}}$ are the elastic, plastic, and thermal strain components, respectively. The elastic strain is obtained from the mechanical constitutive law

(6) $$\sigma =C\varepsilon_{\mathrm{el}},$$

where C is the fourth-order material stiffness tensor. The plastic strain is obtained by imposing the yield criterion

(7) $$f=\sigma_{v}-\sigma_{y}=0$$

on the plastic flow law

(8) $$\varepsilon_{\mathrm{pl}}={\bar{\varepsilon }}_{\mathrm{pl}}\left(\frac{\partial f}{\partial \sigma }\right),$$

where $\sigma_{v}$ is the von-Mises stress, $\sigma_{y}$ is the material-specific and temperature-dependent yield strength, and ${\bar{\varepsilon }}_{\mathrm{pl}}$ denotes the equivalent plastic strain. The thermal strain is calculated using the equation

(9) $$\varepsilon_{\mathrm{th}}=\alpha \left(T-T_{\mathrm{ref}}\right),$$

in which α is the temperature-dependent thermal expansion coefficient and $T_{\mathrm{ref}}$ is the reference temperature.

### 4.2. Boundary Conditions and Material Properties

In the thermal simulation, a convection boundary condition was applied to all surfaces of the half-cylinder shell substrate at the beginning of the simulation. For the deposition geometry, the local boundary conditions in the process zone were updated with each activation of elements. As new elements with free surfaces emerged, they were assigned the convection boundary, while neighboring elements that lost their free surface also lost the convection boundary. In the mechanical simulation, a fixed boundary condition was defined at the boreholes of the substrate to mimic the fixations in the experiment.

**Figure 6:**
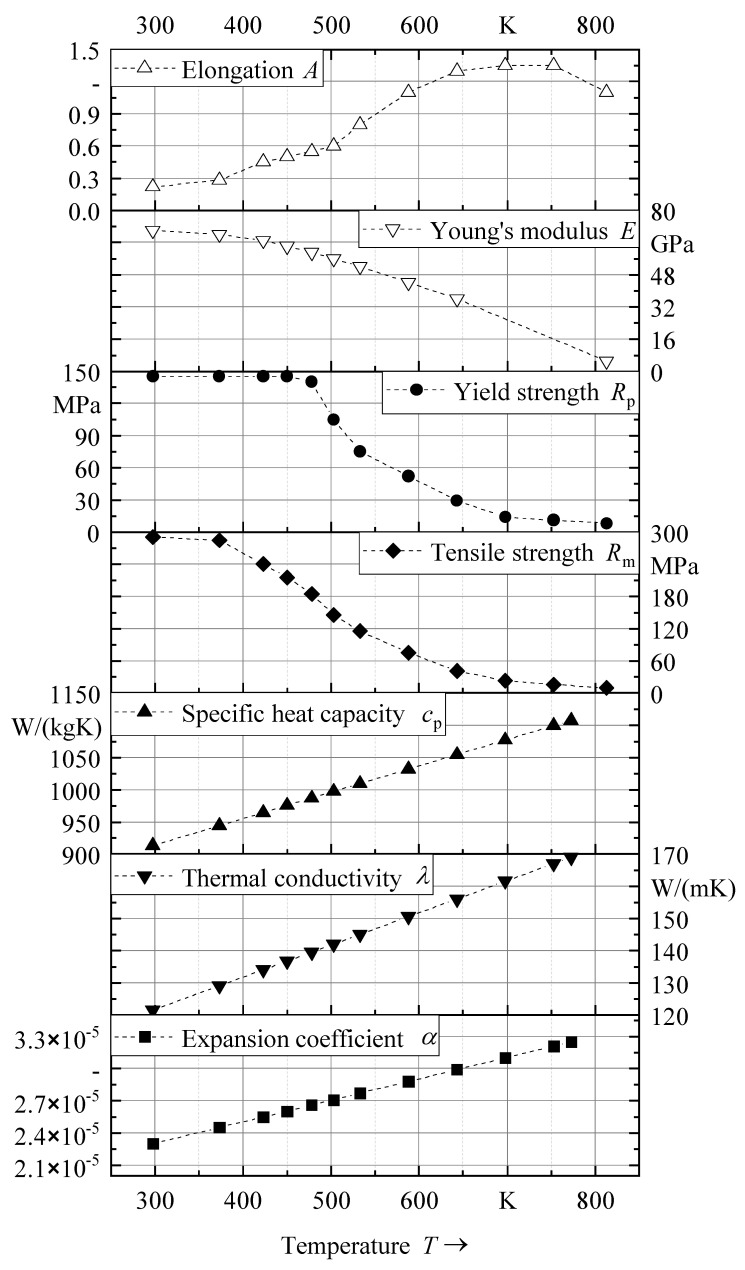
Temperature-dependent material properties of AA 5083 and AA 5183.

The temperature-dependent material properties for the substrate (AA 5083) and the wire (AA 5183) used in the thermo-mechanical simulations were assumed to be equal and were obtained from the literature [20,21]. The density and Poisson’s ratio were assumed to be constant at 2660 kg/m^3^ and 0.33, respectively. Figure 6 illustrates the remaining material properties. Values above the melting temperature were assumed to be constant at the given value closest to the melting temperature.

The simulation required the true stress $\sigma_{t}$ and true strain $\varepsilon_{t}$ values, which were calculated for the plastic section of the materials using the conversion formulas

(10) $$\sigma_{t}=\sigma \left(1+\varepsilon \right)$$

and

(11) $$\varepsilon_{t}=\ln\left(1+\varepsilon \right).$$

Here, σ and ε denote the engineering stress and strain.

### 4.3. Mesh

The parts to be simulated were meshed using linear hexahedral elements. The same mesh was employed for both the thermal and the structural simulation to avoid the necessity of interpolation during coupling. Two linear elements were used to resolve the width and the height of each weld bead. This meshing strategy is based on the mesh convergence study of a previous simulation [16]. By applying this rule to the deposition geometry, the number of required elements could be calculated. The number of elements in axial and in tangential directions was double the number of weld beads of the axial and the tangential deposition patterns, resulting in 20 and 54 elements, respectively. With a maximum of three WAAM layers in the deposition geometry, six elements were used in the through-thickness direction. The half-cylinder shell was resolved using three elements for the specimens with a thickness of 8 mm or 10.5 mm. For specimen thicknesses of 18 mm and 30 mm, the number of elements increased to six and seven, respectively. The element sizes in other parts of the geometry were chosen to roughly equal the element sizes of the shell below the deposition geometry. Table 3 gives the total number of elements for each simulated shell thickness. The additional nodes and elements in the 8 mm shell compared to the 10.5 mm shell stem from the additional rim in the transition area between the thinner shell and the unchanged flange.

### 4.4. Calibration

In the calibration process, the heat source efficiency (η) and the thermal convection coefficient to air (k) were adjusted so that the simulated temperature histories matched the measurements from the experiment. The heat source dimensions were set according to the weld bead dimensions with *h* = 3 mm, *w* = 10 mm, and *l* = 10 mm. The ambient temperature was measured to be 297 K at the start of the experiments and was assumed to be constant for the rest of the simulation. The contact boundary condition between the clamping screws and the part was not individually considered since the affected area was relatively small and located far from the process zone.

**Figure 7:**
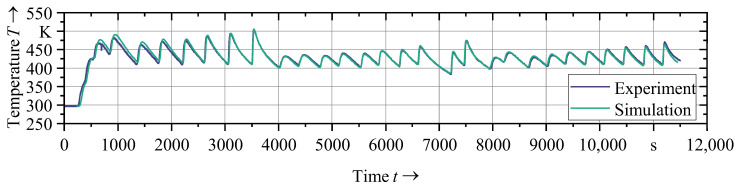
Comparison of thermal measurements and simulation results for the calibrated model at TC2 of the calibration specimen (*d*_s_ = 8 mm, *d*_a_ = 9 mm).

The calibration specimen (*d*_s_ = 8 mm, *d*_a_ = 9 mm) was manufactured using the tangential deposition pattern. The average power value for each weld bead was obtained from the log file of the welding power source. Figure 7 shows the temperature histories of the measurements and of the calibrated model at TC2, with the calibrated parameters given in Table 4. The simulation closely followed the measurements, indicating a successful calibration of the thermal model.

## 5. Results and Discussion

### 5.1. Thermal Validation

The thermal simulation was validated by comparing the experimental results obtained during the WAAM process with the simulated thermal history obtained from the calibrated model.

**Figure 8:**
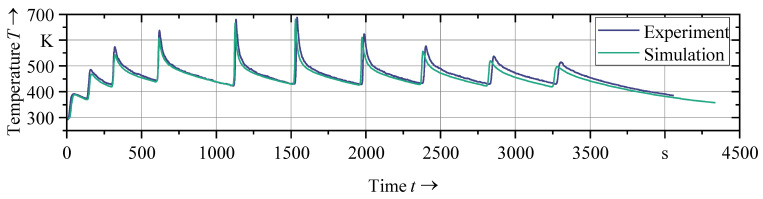
Comparison of thermal measurements and simulation results of the calibrated model at TC2 of the validation specimen (*d_s_* = 10.5 mm, *d_a_* = 3 mm).

The validation specimen (*d*_s_ = 10.5 mm, *d*_a_ = 3 mm) was manufactured using a different shell thickness, and thus allowed the validation of the simulation’s ability to predict the thermal history for shells of varying thicknesses. Figure 8 illustrates the thermal histories of the validation specimen of the experiment and of the simulation at TC1 (Figure 7). The result indicates that the simulation model is a reliable tool for predicting the thermal behavior of WAAM processes, as it predicts temperatures accurately, regardless of variations in the shell thickness and the thermocouple location.

### 5.2. Structural Validation

**Figure 9:**
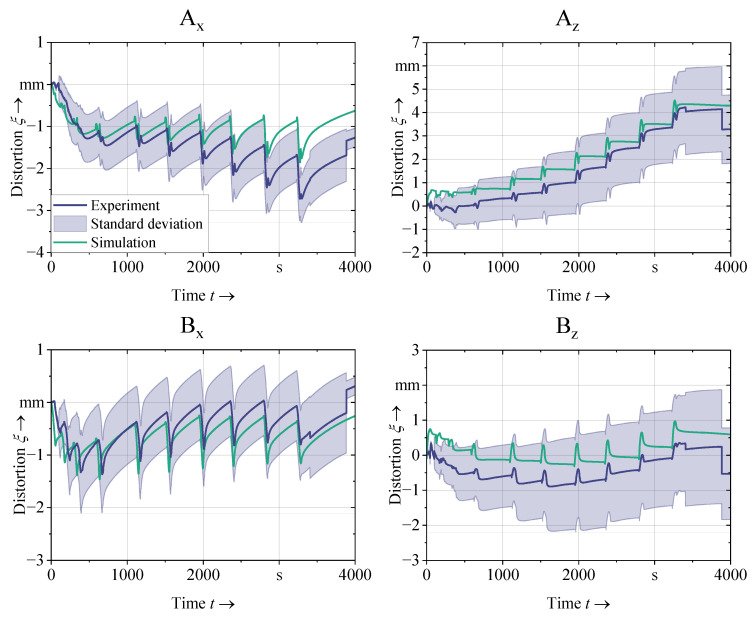
Structural validation of the simulation using single-layer specimens (*d*_s_ = 10.5 mm, *d*_a_ = 3 mm); the standard deviation was calculated from a total of four experimental passes using the same setup.

The structural validation was performed using a similar approach. The distortion history was measured along A_x_, A_z_, B_x_, and B_z_ in four experimental passes of the single-layered specimen (*d*_s_ = 10.5 mm, *d*_a_ = 3 mm) and in one experimental pass of the three-layered specimen (*d*_s_ = 10.5 mm, *d*_a_ = 9 mm). The average distortion history of the single-layered specimens is shown in Figure 9, with the confidence interval indicating the standard deviation ±σ in the positive and the negative direction.

By comparing the measurements to the simulation results, it was observed that the simulation could replicate the trends of each of the measured displacement histories in detail. Furthermore, the simulation results stayed within the confidence interval for A_z_, B_x_, and B_z_. In the case of A_x_, the simulation remained within the confidence interval for most of the simulated time. Toward the end of the measurement duration, occasional jumps can be observed in the distortion histories. This can be attributed to the fact that the distortion exceeded the measuring range of the displacement transducers before the end of the process. The jumps in the data indicate displacement transducers slipping off the edge of the shell geometry during the experiment.

**Figure 10:**
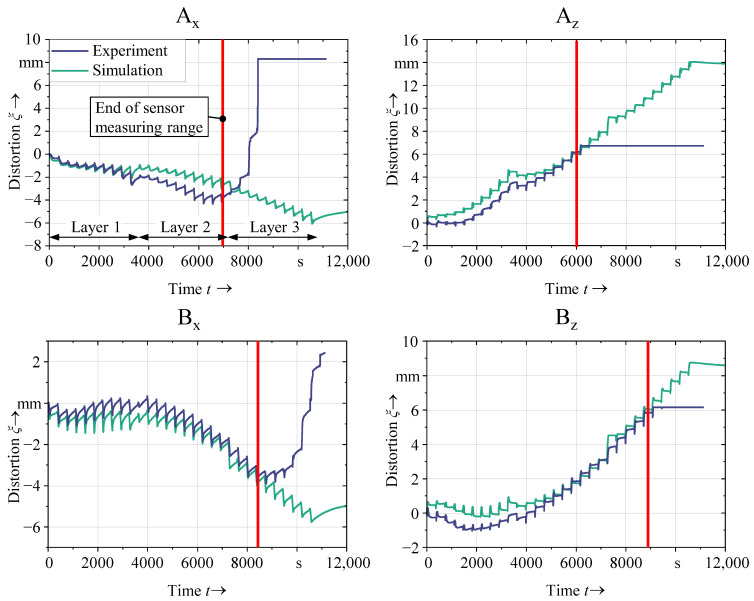
Structural validation of the simulation using a three-layered specimen (*d*_s_ = 10.5 mm, *d*_a_ = 9 mm).

In Figure 10, the simulation result for the three-layered specimen is compared with the measurements. Initially, the simulation matched the experimental results both qualitatively and quantitatively very closely. However, a divergence between the two sets of data became evident during the second half of the simulation, where, again, the displacement transducers reached their limits. Nevertheless, the comparison between the three-layered specimens with the corresponding experiments indicates that the simulation is also valid for larger time scales.

### 5.3. Variation of the Half-Cylinder Shell Thickness

**Figure 11:**
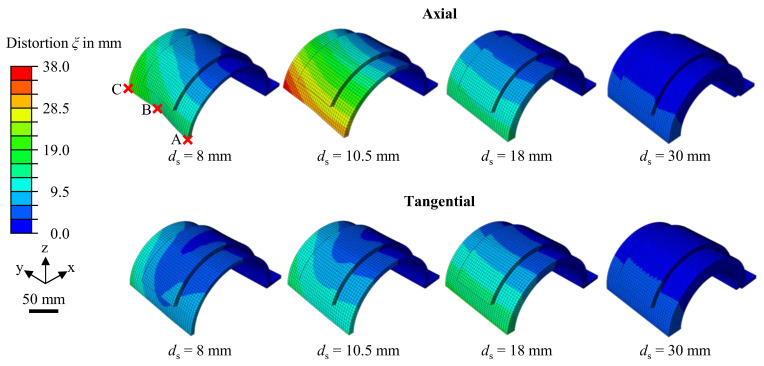
Simulation results for varying shell thicknesses and an axial deposition sequence for a total simulated time of 200 min.

**Figure 12:**
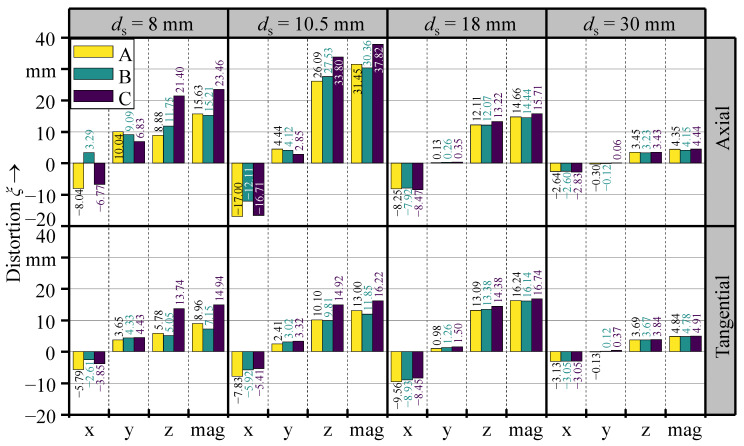
Distortion measured at A, B, and C at the end of the simulations, given in the x-, y-, and z-direction and as their combined magnitude.

With the validated model, the effect of different shell thicknesses on the part distortion was investigated through simulations. The thermo-mechanical simulation was performed for the axial and the tangential deposition pattern for four different shell thicknesses of 8 mm, 10.5 mm, 18 mm, and 30 mm. The deposition geometry consisted of three layers in all simulated variations. Figure 11 illustrates the qualitative simulation results at the end of the WAAM process. At the same time, Figure 12 gives the quantitative distortion for the measuring positions A, B, and C.

According to the simulation results, the maximum distortion magnitude occurred at measuring point C, regardless of the shell thickness or the deposition pattern. The qualitative illustration and the distortion values for measuring points A and C also suggest an asymmetric distortion of the parts in the x–z-plane. The phenomenon of asymmetric distortion in both deposition patterns can be attributed to the progressive heat input and the incremental material deposition during the WAAM process. As a bead was deposited, the substrate material was heated progressively, leading to a gradual reduction of the Young’ modulus and the yield strength, making the material more susceptible to distortion. This effect was more pronounced towards the end of the weld bead. In the case of the axial deposition pattern, the weld beads were individually applied in the positive y-direction and successively deposited in the circumferential direction. This operating sequence encouraged higher distortion values at measuring point C, as it was positioned closer to the end of the weld beads. An additional effect needs to be considered in the case of the tangential deposition pattern, in which the weld beads were individually deposited in the circumferential direction and successively applied in the positive y-direction. The area moment of inertia in the y–z-plane rose incrementally after each deposited weld bead by the effect of the added cross-sectional area of the weld bead. This increased the stiffness of the half-cylinder shell geometry incrementally in the positive y-direction throughout the manufacturing of each layer. Hence, this deposition pattern also encouraged a higher distortion in the proximity of measuring point C. Increasing the shell thickness naturally led to a faster dissipation of the introduced heat and also to a higher shell stiffness. Therefore, the impact of progressive heat input and incremental material deposition decreased. This correlation is evident in the distortion iso-contours depicted in Figure 11, where straighter lines indicate a more symmetric distortion. Additionally, Figure 12 shows a decrease in the variance of measured distortion values at points A, B, and C in each direction as the shell thickness increases.

No continuous relation between the shell thickness and the distortion was observed. The simulation with a shell thickness of *d*_s_ = 10.5 mm and an axial deposition pattern resulted in the highest distortion magnitude, reaching 37.82 mm. Regarding the tangential deposition pattern, the most significant distortion was observed in the simulation with a shell thickness of *d*_s_ = 18 mm, peaking at 16.74 mm, which is less than half the magnitude compared to the axial deposition pattern. The lowest distortion values were measured in the specimens with the highest shell thickness of *d*_s_ = 30 mm for both investigated deposition patterns.

**Figure 13:**
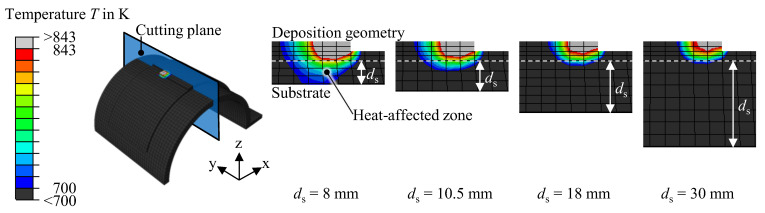
Resulting heat affected zone when using the axial deposition pattern for different shell thicknesses at the simulation time t = 5388 s.

The effect of the half-cylinder shell thickness on the distortion seen in the simulated specimens can be explained by the interplay of two factors. On the one hand, a thick shell leads to higher temperature gradients during the welding process due to the introduced heat dissipating quickly inside the large substrate volume. Steep temperature gradients drive the formation of high residual stresses [5,22,23]. Thus, thick shells favor the formation of distortions in this regard. On the other hand, a thick shell is more resistant to distortion due to its inherent higher stiffness. Figure 13 shows the dimensions of the heat-affected zone relative to the shell thickness. The temperature scale utilized for the figure ranges from 843 K, the melting temperature, down to 700 K, a temperature at which the material experiences a reduction of approximately 73% in the Young’s modulus and 90% in the yield strength (see Figure 6), as compared to the values at room temperature. Furthermore, as the shell thickness increased, the dimensions and the penetration depth of the heat-affected zone decreased, with full penetration of the substrate occurring only at a shell thickness of *d*_s_ = 8 mm. Deep penetration of the heat-affected zone into the substrate softens the material, and thus increases its susceptibility to distortions. However, the large size of the heat-affected zone also indicates lower temperature gradients, which disfavor the formation of residual stresses. These two competing effects lead to distortion maxima in the investigated simulations with a shell thickness of 10.5 mm and 18 mm for the axial and the tangential deposition patterns, respectively.

When the investigated deposition patterns are compared, the axial deposition pattern was associated with a significantly higher distortion for thinner shells (*d*_s_ = 8 mm and *d*_s_ = 10.5 mm), while the axial and the tangential deposition pattern exhibited similar distortion values when applied to thicker shells (*d*_s_ = 18 mm and *d*_s_ = 30 mm).

The influence of the deposition pattern on the distortion magnitude can be attributed to differences in the heat propagation inside the parts. The local temperature gradients are greater in the transverse direction of the weld bead than in the longitudinal direction [6,15]. Hence, the formation of residual stresses is expected to be higher in the transverse direction of the beads. In the case of the axial deposition pattern, the transverse residual stresses of each weld bead are combined, and thus bend the half-cylinder, increasing its diameter. Additionally, the area moment of inertia is low due to the rectangular shape of the substrate cross-section in the rotational plane of the cylinder. By contrast, the transverse residual stresses from the weld beads of the tangential deposition pattern compound to indent the half-cylinder shell towards its center axis. This effect is visible in Figure 11, by the rounded distortion iso-contours for the respective specimens with a shell thickness of *d*_s_ = 8 mm and *d*_s_ = 10.5 mm. In this case, the bending is impeded by a higher area moment of inertia due to the cut area of the substrate geometry in the y–z-plane. The effects of weld bead orientation and area moment of inertia have a greater impact on thin shells, as the axial deposition pattern produced a higher distortion for shell thicknesses of *d*_s_ = 8 mm and *d*_s_ = 10.5 mm. However, these effects diminish as the shell thickness increases further and the shell becomes stiffer.

## 6. Conclusions and Outlook

This study investigated the effects of different substrate thicknesses and deposition patterns on the part distortion of a WAAM-reinforced half-cylinder shell geometry. Measurements were used to calibrate and validate a thermo-mechanical simulation, which was then employed to simulate different configurations for the substrate and the deposition pattern. The key findings can be summarized as follows:

1. For the same deposition geometry, the shell thickness and the deposition pattern have a significant impact on the part distortion.
2. The maximum distortion magnitude occurred at measuring point C in all simulations. An asymmetric distortion of the parts in the x–z-plane was observed due to the progressive heat input and the incremental material deposition during the WAAM process in both investigated deposition patterns. The impact of those effects decreased with increasing shell thicknesses.
3. The relation between the shell thickness and the distortion was non-linear and results from the interplay of two factors. As the shell thickness increases, both the resistance to bending and the formation of residual stresses increase due to the steeper thermal gradients. Amongst the investigated specimens, the highest distortion magnitude was observed in the simulation with a medium shell thickness of 10.5 mm and an axial deposition pattern.
4. While the axial deposition pattern was associated with a significantly higher distortion for thinner shells, the axial and the tangential deposition pattern exhibited similar distortion values when applied to thicker shells.

This study only examined two basic deposition patterns and four different half-cylinder shell thicknesses. While these findings shed light on the behavior of the studied configurations, further research could explore more complex deposition patterns in combination with other relevant substrate geometries. Such investigations could lead to the development of optimized strategies to reduce distortions in specific critical regions. Moreover, the simulation relied on material properties obtained from the literature. To further enhance the accuracy of the simulation, hot tensile tests could be conducted on the material in order to obtain more precise data.

## Figures and Tables

**Table 1:** Welding parameters used in the experiments.

Welding Process	Current in A	Voltage in V	Wire Feed Speed in mm/s
Fronius PMC Mix (first layer)	138	19.6	13.3
Fronius CMT (other layers)	123	14.6	13.3

**Table 2:** Chemical compositions of the wire and substrate material (ratios given in m.%).

Material	Si	Cu	Fe	Mn	Mg	Cr	Ti	Zn	Al
AA 5183 (wire)	0.04	<0.01	0.16	0.63	4.94	0.08	0.10	0.25	balance
AA 5083 (substrate)	0.40	0.10	0.40	<1.00	<4.90	<0.25	0.15	0.25	balance

**Table 3:** Mesh characteristics.

*d*_s_ in mm	Total Number of Elements	Total Number of Nodes
8	16,152	20,374
10.5	15,282	19,346
18	24,272	28,804
30	25,512	29,994

**Table 4:** List of the calibrated simulation parameters.

Parameter	Value
Heat source height h	3 mm
Heat source width *w*	10 mm
Heat source length *l*	10 mm
Heat source efficiency *η*	0.8
Convection coefficient to air *k*	13 W/(m^2^K)

## Data Availability

Not applicable.

## References

[1] Ding D., Pan Z., Cuiuri D., Li H. (2015). Wire-feed additive manufacturing of metal components: Technologies, developments and future interests. Int. J. Adv. Manuf. Technol..

[2] Wu B., Pan Z., Ding D., Cuiuri D., Li H., Xu J., Norrish J. (2018). A review of the wire arc additive manufacturing of metals: Properties, defects and quality improvement. J. Manuf. Process..

[3] (2021). Unfired Pressure Vessels—Part 3: Design.

[4] (2023). Water-Tube Boilers and Auxiliary Installations—Part 3: Design and Calculation for Pressure Parts of the Boiler.

[5] Vasinonta A., Beuth J.L., Griffith M. (2007). Process Maps for Predicting Residual Stress and Melt Pool Size in the Laser-Based Fabrication of Thin-Walled Structures. J. Manuf. Sci. Eng..

[6] Radaj D. (1992). Heat Effects of Welding: Temperature Field, Residual Stress, Distortion.

[7] Belitzki A., Stadter C., Zaeh M.F. (2019). Distortion minimization of laser beam welded components by the use of finite element simulation and Artificial Intelligence. CIRP J. Manuf. Sci. Technol..

[8] Biswas P., Mandal N.R., Das S. (2011). Prediction of welding deformations of large stiffened panels using average plastic strain method. Sci. Technol. Weld. Join..

[9] Li X., Lin J., Xia Z., Zhang Y., Fu H. (2021). Influence of Deposition Patterns on Distortion of H13 Steel by Wire-Arc Additive Manufacturing. Metals.

[10] Israr R., Buhl J.E., Elze L., Bambach M. Simulation of different path strategies for wire-arc additive manufacturing with Lagrangian finite element methods. Proceedings of the LS-Dyna Forum 2018.

[11] Wu Q., Mukherjee T., Liu C., Lu J., DebRoy T. (2019). Residual stresses and distortion in the patterned printing of titanium and nickel alloys. Addit. Manuf..

[12] Ahmad B., Zhang X., Guo H., Fitzpatrick M.E., Neto L.M.S.C., Williams S. (2022). Influence of Deposition Strategies on Residual Stress in Wire + Arc Additive Manufactured Titanium Ti-6Al-4V. Metals.

[13] Lu X., Lin X., Chiumenti M., Cervera M., Hu Y., Ji X., Ma L., Yang H., Huang W. (2019). Residual stress and distortion of rectangular and S-shaped Ti-6Al-4V parts by Directed Energy Deposition: Modelling and experimental calibration. Addit. Manuf..

[14] Hesse W. (2016). Aluminium-Werkstoff-Datenblätter: Aluminium Material Data Sheets.

[15] Lindgren L.E. (2007). Computational Welding Mechanics: Thermomechanical and Microstructural Simulations.

[16] Zhao X.F., Wimmer A., Zaeh M.F. (2023). Experimental and simulative investigation of welding sequences on thermally induced distortions in wire arc additive manufacturing. Rapid Prototyp. J..

[17] Michaleris P. (2014). Modeling metal deposition in heat transfer analyses of additive manufacturing processes. Finite Elem. Anal. Des..

[18] Goldak J., Chakravarti A., Bibby M. (1984). A new finite element model for welding heat sources. Met. Trans B.

[19] Zapata A., Zhao X.F., Li S., Bernauer C., Zaeh M.F. (2023). Three-dimensional annular heat source for the thermal simulation of coaxial laser metal deposition with wire. J. Laser Appl..

[20] Kaufman J.G., Kaufman J.G. (1999). Properties of Aluminum Alloys: Tensile, Creep, and Fatigue Data at High and Low Temperatures.

[21] (2007). Eurocode 9: Design of Aluminium Structures—Part 1–2: Structural Fire Design.

[22] Mercelis P., Kruth J.-P. (2006). Residual stresses in selective laser sintering and selective laser melting. Rapid Prototyp. J..

[23] Li C., Liu Z.Y., Fang X.Y., Guo Y.B. (2018). Residual Stress in Metal Additive Manufacturing. Procedia CIRP.

